# SARS-CoV-2 in animals: potential for unknown reservoir hosts and public health implications

**DOI:** 10.1080/01652176.2021.1921311

**Published:** 2021-05-14

**Authors:** Khan Sharun, Kuldeep Dhama, Abhijit M. Pawde, Christian Gortázar, Ruchi Tiwari, D. Katterine Bonilla-Aldana, Alfonso J. Rodriguez-Morales, José de la Fuente, Izabela Michalak, Youssef A. Attia

**Affiliations:** a Division of Surgery, ICAR-Indian Veterinary Research Institute, Bareilly, India; b Division of Pathology, ICAR-Indian Veterinary Research Institute, Bareilly, India; c SaBio IREC Instituto de Investigación en Recursos Cinegéticos (CSIC-Universidad de Castilla-La Mancha), Ciudad Real, Spain; d Department of Veterinary Microbiology and Immunology, College of Veterinary Sciences, Uttar Pradesh Pandit Deen Dayal Upadhyaya Pashu Chikitsa Vigyan Vishwavidyalaya Evam Go Anusandhan Sansthan (DUVASU), Mathura, India; e Semillero de Investigación en Zoonosis (SIZOO), Grupo de Investigacion BIOECOS, Fundacion Universitaria Autonoma de las Americas, Pereira, Colombia; f Faculty of Health Sciences, Public Health and Infection Research Group, Universidad Tecnologica de Pereira, Pereira, Colombia; g Faculty of Medicine, Grupo de Investigacion Biomedicina, Fundacion Universitaria Autonoma de las Americas, Pereira, Colombia; h Latin American Network of Coronavirus Disease 2019-COVID-19 Research (LANCOVID-19), Pereira, Colombia; i School of Medicine, Universidad Privada Franz Tamayo, (UNIFRANZ), Cochabamba, Bolivia; j Department of Veterinary Pathobiology, Center for Veterinary Health Sciences, Oklahoma State University, Stillwater, OK, USA; k Faculty of Chemistry, Department of Advanced Material Technologies, Wroclaw University of Science and Technology, Wroclaw, Poland; l Faculty of Environmental Sciences, Department of Agriculture, King Abdulaziz University, Jeddah, Saudi Arabia; m The Strategic Center to Kingdom Vision Realization, King Abdulaziz University, Jeddah, Saudi Arabia; n Faculty of Agriculture, Animal and Poultry Production Department, Damanhour University, Damanhour, Egypt

**Keywords:** Animals, host range, COVID-19, wildlife reservoir, SARS-CoV-2, susceptibility, public health

## Abstract

Severe acute respiratory syndrome coronavirus 2 (SARS-CoV-2, previously 2019-nCoV) is suspected of having originated in 2019 in China from a coronavirus infected bat of the genus *Rhinolophus*. Following the initial emergence, possibly facilitated by a mammalian bridge host, SARS-CoV-2 is currently transmitted across the globe via efficient human-to-human transmission. Results obtained from experimental studies indicate that animal species such as cats, ferrets, raccoon dogs, cynomolgus macaques, rhesus macaques, white-tailed deer, rabbits, Egyptian fruit bats, and Syrian hamsters are susceptible to SARS-CoV-2 infection, and that cat-to-cat and ferret-to-ferret transmission can take place via contact and air. However, natural infections of SARS-CoV-2 have been reported only in pet dogs and cats, tigers, lions, snow leopards, pumas, and gorillas at zoos, and farmed mink and ferrets. Even though human-to-animal spillover has been reported at several instances, SARS-CoV-2 transmission from animals-to-humans has only been reported from mink-to-humans in mink farms. Following the rapid transmission of SARS-CoV-2 within the mink population, a new mink-associated SARS-CoV-2 variant emerged that was identified in both humans and mink. The increasing reports of SARS-CoV-2 in carnivores indicate the higher susceptibility of animal species belonging to this order. The sporadic reports of SARS-CoV-2 infection in domestic and wild animal species require further investigation to determine if SARS-CoV-2 or related Betacoronaviruses can get established in kept, feral or wild animal populations, which may eventually act as viral reservoirs. This review analyzes the current evidence of SARS-CoV-2 natural infection in domestic and wild animal species and their possible implications on public health.

## 1. Introduction

Coronaviruses (CoVs) are reported to cause diseases in a wide range of hosts, including humans and animals. Among the different CoVs, three recently emerged coronavirus were reported to be zoonotic (Rodriguez-Morales et al. 2020a). They are the Severe Acute Respiratory Syndrome CoV (SARS-CoV), Middle East Respiratory Syndrome CoV (MERS-CoV) and the recent Severe Acute Respiratory Syndrome CoV 2 (SARS-CoV-2) that originated in Wuhan, China, causing coronavirus disease 2019 (COVID-19) (Malik et al. 2020; Rabaan et al. 2020; Sharun 2020). Compared to the previous zoonotic CoVs, SARS-CoV-2 is less pathogenic but more transmissible, which is evident from the growing number of COVID-19 cases globally due to efficient human-to-human transmission (Dhama et al. 2020a). The consequences of COVID-19 pandemic affect all aspects of human activity, including animal health and posed severe adverse socio-economic impacts worldwide (Ahmad et al. 2020; Ciotti et al. 2020; Gortázar and de la Fuente 2020; Nicola et al. 2020). SARS-CoV-2 is suspected to be originated from the Huanan South China Seafood Market, a live-animal market in Wuhan, China (Tiwari et al. 2020; Zhang and Holmes 2020). This seafood and wildlife market is reported to have housed a variety of mammalian species (Zhang and Holmes 2020). Such live-animal markets provide ideal conditions that promote the inter-species contact between wild and domesticated animal species, and human beings. Following the transmission to a bridge host, the SARS-CoV-2 might have undergone adaptive genetic recombination that facilitated its transmission to human beings (Ghosh and Malik 2020; Tiwari et al. 2020).

Both SARS-CoV and MERS-CoV have bats as the original host (Zhang and Holmes 2020). The close genetic similarity existing between SARS-CoV-2 and a coronavirus isolated from the intermediate horseshoe bats (*Rhinolophus affinis*) suggest that this novel virus might also have originated from bats (Mallapaty 2020a). However, there is a small difference (4%) in the genetic makeup of the SARS-CoV-2 from the closest bat-CoV identified (Li et al. 2020a; Mallapaty 2020a) indicating the possibility of a bridge host that facilitated the species jump from bats to human beings. The hosts that played an essential role in the transmission of CoV from bats to humans in the case of SARS and MERS outbreaks are palm civets (*Paradoxurus hermaphroditus*) and dromedary camels (*Camelus dromedarius*), respectively (Zhang and Holmes 2020). Pangolins (*Manis* sp) were considered as the primary suspect that could act as a bridge host for SARS-CoV-2 (Mallapaty 2020a). SARS-CoV-2-related CoVs were isolated from Malayan pangolins (*Manis javanica*) that were seized in southern China (Lam et al. 2020). However, the actual bridge host remains unknown.

**Figure 1.**
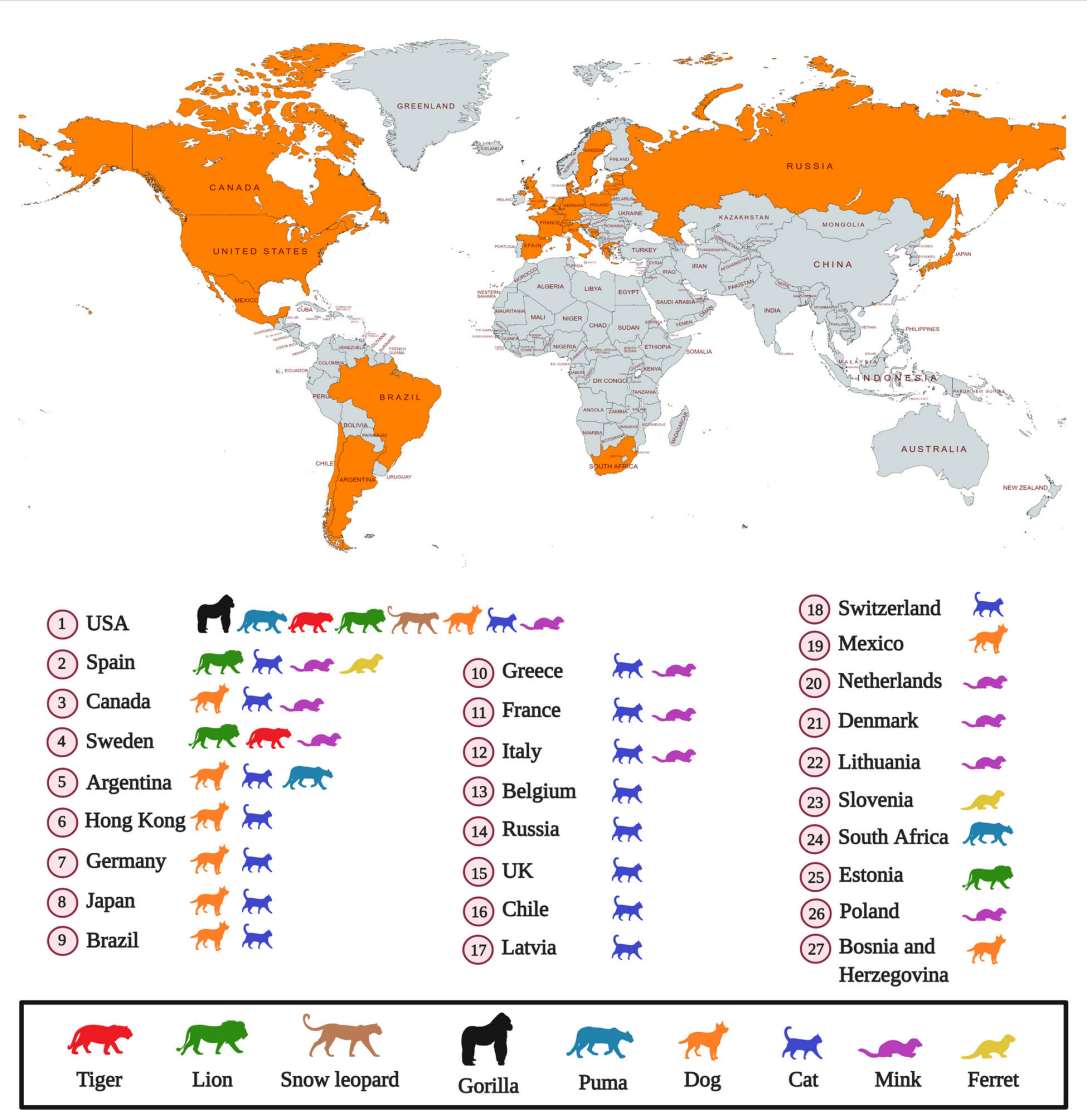
Reports of SARS-CoV-2 natural infection in animals as on March 29, 2021. Data collected from OIE (World Organisation for Animal Health, COVID-19 Portal, Events in animals, Available at: https://www.oie.int/en/scientific-expertise/specific-information-and-recommendations/questions-and-answers-on-2019novel-coronavirus/events-in-animals/).

Several reports of SARS-CoV-2 infection in different animals, wildlife species and experimental animal models have been published, and the current dynamics of the virus affecting humans as COVID-19 pandemic necessitates further detailed investigations concerning the transmission ability of the virus from humans to animals and vice versa and enhancing implementation of one health approach (Delahay et al. 2020; Franklin and Bevins 2020; Leroy et al. 2020; Martínez-Hernández et al. 2020; Salajegheh Tazerji et al. 2020; Sharun et al. 2020a; Tiwari et al. 2020). Exploring more research and investigations with regards to the SARS-CoV-2 circulation in animals and its animal-human interface implications along with targeted surveillance and dynamic risk assessments would aid to design and implement effective preventive strategies to limit the transmission of this pandemic virus. Experimental inoculation has identified cats (*Felis catus*), ferrets (*Mustela putorius furo*), raccoon dogs (*Nyctereutes porcyonoides*), hamsters, rhesus macaques (*Macaca mulatta*), white-tailed deer (*Odocoileus virginianus*), rabbits (*Oryctolagus cuniculus*), and fruit bats (*Rousettus aegyptiacus*) to be susceptible to SARS-CoV-2 (Freuling et al. 2020; Muñoz-Fontela et al. 2020; Shi et al. 2020; Younes et al. 2020; Mallapaty 2020a). Natural infections of SARS-CoV-2 have been reported in pet dogs (*Canis lupus familiaris*) and cats, tigers (*Panthera tigris*), snow leopards (*Panthera uncia*), pumas (*Puma concolor*), gorillas (*Gorilla gorilla*), and lions (*Panthera leo*) at zoos, and farmed mink (*Neovison vison*) and ferrets (*Mustela putorius furo*) (Figure 1) (Hobbs and Reid 2020; Jo et al. 2020; Mallapaty 2020a; Gortazar et al. 2021). It is necessary to identify those animal species that are susceptible to SARS-CoV-2 infection since they may act as bridge hosts or virus reservoirs and transmit the infection to human beings. SARS-CoV-2 has already spread extensively among the human population. Therefore, the detection of this virus in domestic or wild animals may not necessarily confirm their role as reservoirs or as bridge hosts. That is because such occurrences might be the result of disease spillover from humans to animals (Mallapaty 2020a). This review analyzes the current scenario of SARS-CoV-2 infection in different animal species including wild animal species and their public health implications.

## 2. Host range of SARS-CoV-2

Coronaviruses are known for their capacity to jump the so-called “species barrier” that facilitates the transmission of pathogens between different species. This inherent genetic plasticity enables the CoVs to have a broad host range (Zhang and Holmes 2020; Dhama et al. 2020b). A unique peptide (PRRA) insertion was identified in the human SARS-CoV-2 virus at the S1 and S2 junction of the spike protein (S). This insertion might be involved in the proteolytic cleavage of the S protein and could have an impact on the host range as well as transmissibility (Li et al. 2020b). The effective binding of SARS-CoV-2 S protein to the host receptor angiotensin-converting enzyme 2 (ACE2) is one of the critical prerequisites for infecting a particular species and therefore determines the host range (Zhai et al. 2020). In other words, a species that possess human-like ACE2 receptor is at risk of getting infected with SARS-CoV-2 (Wei et al. 2021). Understanding the conservation of ACE2 receptor and its expression pattern across different animal species may provide insight into the potential hosts of SARS-CoV-2 (Sun et al. 2020). Based on the ACE2 homology studies, the direct transmission of SARS-CoV-2 from bat to human beings are unlikely. However, the rapid adaptation of a bat SARS-like-CoV in different high-risk mammalian species that shares good ACE2 homology with human beings might allow the virus to acquire high binding potential with human-like ACE2 receptors (Wei et al. 2021).

ACE2 genes of several mammalian species were found to be highly conserved, suggesting that SARS-CoV-2 can potentially bind to the ACE2 proteins of a broad range of animal species (Sun et al. 2020). In addition to that, cats and dogs were found to be the species that are closest to human beings (among rat, mouse, hamster, rabbit, bat, sheep, goat, cattle, pig, dog, and cat) in the phylogenetic tree plotted based on the ACE2 proteins (Sun et al. 2020). Furthermore, Sun et al. (2020) studied the expression pattern of ACE2 in different animal species. The ACE2 gene was found to express in tissues such as kidney and liver highly (Sun et al. 2020). It was also found to be expressed in the skin, lungs, ear tip, and retina in case of cats while skin and retina in case of dogs. It was also interesting to note that both cats and ferrets expressed ACE2 in the lungs, therefore may act as an ideal model for studying COVID-19.

It was found that the species known to lack susceptibility to SARS-CoV-2 infection have non-conservative mutations in several ACE2 amino acid residues. This mutation will disrupt the key polar and charged contacts with SARS-CoV-2 S protein (Rodrigues et al. 2020). Among the 20 amino acids present in ACE2 receptor that come in contact with the viral S protein, ACE2 can still act as the receptor of SARS-CoV-2 even if seven of the amino acids are replaced (Zhai et al. 2020). Initially, snakes and turtles were also considered as possible bridge hosts of SARS-CoV-2. However, almost half of the critical residues in ACE2 that are required for associating with the receptor-binding domain (RBD) of SARS-CoV-2 S protein were changed (Luan et al. 2020a). Therefore, snakes and turtles cannot act as the bridge hosts for SARS-CoV-2.

The critical amino acids present in ACE2 were compared among different species to evaluate the ability to bind the RBD of SARS-CoV-2 S protein. It was found that the ACE2 proteins from Primates, Cricetidae, Bovidae, and Cetacea can recognize the SARS-CoV-2 RBD since they maintain the majority of the critical residues in ACE2 (Luan et al. 2020a). Further analysis using the structural simulation of RBD and ACE2 found that the ACE2 proteins from Bovidae and Cricetidae can associate with SARS-CoV-2 RBD (Luan et al. 2020a). Therefore, it was suggested to conduct further investigation on animals of the *Bovidae* and *Cricetidae* family to identify the potential bridge host of SARS-CoV-2.

Other than the ACE2 receptor, TMPRSS2 has a role in the entry of SARS-CoV-2 into the host cell. The virus uses it for priming the S protein, thereby facilitating cell entry (Matsuyama et al. 2020; Schmitt et al. 2020). Genetic analysis of the ACE2 and TMPRSS2 sequence has suggested savanna monkeys (*Chlorocebus* sp.) to be susceptible to hosts for SARS-CoV-2 infection. The study also pointed out the possibility of bi-directional infections between human beings and savanna monkeys (Schmitt et al. 2020).

The ACE2 sequences of 410 vertebrate species (252 mammals, 72 birds, 65 fishes, 17 reptiles and four amphibians) were compared to predict their ability to bind SARS-CoV-2 RBD (Damas et al. 2020). The interaction between ACE2 and RBD were classified into five categories: very high, high, medium, low, and very low. A total of 18 animal species came under the category of “very high”, all belonging to Old World primates and apes that exhibited complete similarity to humans across all the 25 ACE2 binding residues. Another 28 species came under the “high” category (high likelihood of binding RBD). This includes species from cetaceans (12), rodents (7), cervids (3), lemuriform primates (3), along with Giant anteater (*Myrmecophaga tridactyla*), Southern tamandua (*Tamandua tetradactyla*), and Angola colobus (*Colobus angolensis*) (Damas et al. 2020). Although *in silico* studies suggest a broad host range for SARS-CoV-2, further validation is required using experimental inoculation before confirming the susceptibility.

## 3. SARS-CoV-2-related CoVs

SARS-CoV-2 enters the host cell through the interaction of its S protein (RBD) with the host ACE2 receptor (Wan et al. 2020). Only two mammals have been reported to harbour SARS-CoV-2-related CoVs. They are pangolins and bats (Lam et al. 2020). The genomic analysis identified SARS-CoV-2 to be closely related to CoVs derived from wild animals, including *Paguma larvata* (Masked palm civet), *Paradoxurus hermaphroditus* (Asian palm civet), *Aselliscus stoliczkanus* (Stoliczka's trident bat), and *Rhinolophus sinicus* (Chinese rufous horseshoe bat). They were present in the same branch of the phylogenetic tree as that of SARS-CoV-2 (Li et al. 2020a). However, SARS-CoV was found to have the highest homology with the Bat coronavirus isolate RaTG13 from *Rhinolophus affinis* (Intermediate Horseshoe Bat) in the whole genome (93.7%), S protein (92.86%), ORF1ab (96.5%), and nucleocapsid protein (96.9%) genes (Li et al. 2020a). Comparative genomic analysis of SARS-CoV-2 with the pangolin-CoV that was isolated from Malayan pangolin identified superior amino acid identity in the E (100%), M (98.6%), N (97.8%), and S (90.7%) proteins. In addition to this, the RBD of the pangolin-CoV S protein is almost identical to SARS-CoV-2 RBD, with only one difference in non-critical amino acid (Xiao et al. 2020). However, in another study, the possibility of SARS-CoV-2 evolving directly from the pangolin-CoV was rejected. They are instead proposing that pangolins may act as natural hosts of Betacoronaviruses that are having the unknown potential to infect human beings (Liu et al. 2020) or have the potential to interfere with diagnostic tests for SARS-CoV-2.

Comprehensive sequence analysis was performed to identify the possible viral reservoir of SARS-CoV-2 in conjunction with relative synonymous codon usage (RSCU) bias among several animal species. Findings obtained from the study suggest that the emergence of SARS-CoV-2 is a result of recombination events between the bat CoV and an unknown CoV at the viral S protein region (Ji et al. 2020). Comparison of the S protein RBD of SARS-CoV-2 with the Bat-CoV-RaTG13 and Pangolin-CoV identified the occurrence of only one substitution in the Pangolin-CoV. In contrast, the Bat-CoV-RaTG13 had five substitutions (Zhai et al. 2020). However, genome analysis of the Pangolin-CoV identified the absence of a furin cleavage motif (RRAR), a unique feature of SARS-CoV-2. Therefore, the possibility for human SARS-CoV-2 virus to be directly originated from pangolins is low (Li et al. 2020b). However, a recent study has identified serological and molecular evidence of novel SARS-CoV-2-related CoVs actively circulating in bats from Eastern Thailand (Wacharapluesadee et al. 2021). The samples collected from five independent bats (*Rhinolophus acuminatus*) yielded a single isolate (RacCS203) that exhibited close relationship with another isolate (RmYN02) found in *Rhinolophus malayanus* (Yunnan, China). The study also detected the presence of highly specific SARS-CoV-2 neutralizing antibodies in the bats sampled from the same colony and in a Malayan pangolin from a wildlife checkpoint in Southern Thailand (Wacharapluesadee et al. 2021). Although the RBD of RacCS203 and RmYN02 failed to bind with ACE2, the antisera raised against the RmYN02 RBD was able to cross-neutralize with SARS-CoV-2 (Wacharapluesadee et al. 2021). These findings reignited the discussion on the possible role played by bats and pangolins in the emergence of SARS-CoV-2. Further studies are required to characterize better the existing CoV population present in bats, pangolins, and other wild animals that could pose a threat in the future.

## 4. Bat coronaviruses (Bat-CoV)

Bats are considered to be the major natural reservoir host of animal and human CoVs. Among the different bat species, the horseshoe bat belonging to the genus Rhinolophus is considered as the reservoir of SARS-like-CoVs (Watanabe et al. 2010). Several novel SARS-CoV-like coronaviruses are continuously being identified and reported in bats all around the world, especially in China (He et al. 2014). These SARS-like-CoVs originating from bats can get subsequently transmitted to humans due to the high mutation rates of CoV as the virus evolves (Kim et al. 2019). The close genomic similarity existing between the bat coronaviruses and SARS-CoV-2 indicate the possibility that this novel virus might have originated from bats similar to its predecessors, MERS-CoV and SARS-CoV (Zhou et al. 2020). Furthermore, genomic analysis of CoVs also indicates the possibility that SARS-CoV-2 is a recombinant virus having characteristics of bat-CoV and another unknown coronavirus (Ji et al. 2020). It was also found that a mutation in the N protein and S protein of SARS-CoV-2 made it distinct from the SARS-like CoV isolated from bats further supporting the hypothesis that SARS-CoV-2 originated from bats after acquiring ability to infect human beings following a series of mutations (Benvenuto et al. 2020). The available evidence indicates that the horseshoe bats belonging to the genus Rhinolophus (*Rhinolophus affinis*) is the natural reservoir host (do Vale et al. 2021).

## 5. Pangolin coronaviruses (Pangolin-CoV)

Pangolin is an animal species that is widely trafficked for their meat and their scales, which are used in Traditional Chinese Medicine. Some of the pangolin species are now threatened with extinction owing to the illegal trading of wild populations (Wang et al. 2020a). Coronavirus have been previously isolated from sick Malayan pangolins that were smuggled for black market trade and were named pangolin-CoV (Liu et al. 2020). In addition to the bat-CoV (RaTG13), the pangolin-CoV (MP789) isolate exhibited strong genetic resemblance to SARS-CoV-2 than any other CoVs isolated from pangolins (Dimonaco et al. 2020; Touati et al. 2020). This resemblance was evident in both gene-level codon usage profiling and whole-genome analysis (Dimonaco et al. 2020). It has been hypothesized that the RBD of SARS-CoV-2 S protein is a hybrid of the bat-CoV (RaTG13) and pangolin-CoV (MP789). The SARS-CoV-2 is believed to have acquired it from the pangolin-CoV via recombination with an ancestral CoV having bat-CoV (RaTG13) genomic background (Flores-Alanis et al. 2020).

## 6. SARS-CoV-2 in domestic animals and feral carnivores

Experimental inoculation of SARS-CoV-2 was performed via the intranasal route to evaluate the susceptibility of several domestic animals. The results indicate that both cats and ferrets are highly susceptible to SARS-CoV-2, while dogs have low susceptibility (Shi et al. 2020). Raccoon dogs were previously considered as bridge hosts for SARS coronavirus in 2002–2004 and have recently been proposed as bridge hosts for SARS-CoV-2, too, after demonstrating susceptibility to infection and transmission to in-contact animals (Freuling et al. 2020). However, chicken, ducks, and pigs were found to lack susceptibility or be of low susceptibility to this novel virus (Meekins et al. 2020; Schlottau et al. 2020; Shi et al. 2020). The lower susceptibility of dogs and pigs to SARS-CoV-2 infection might be due to the relatively low ACE2 receptor expression in their respiratory tract (Zhai et al. 2020). On the contrary, 8-week-old crossbred pigs (*Sus scrufa domesticus*) were found to be susceptible to SARS-CoV-2 infection following oronasal inoculation. Although SARS-CoV-2 RNA was detected in the oral fluids and nasal wash collected from two pigs, live virus was isolated only from one animal. Furthermore, SARS-CoV-2 neutralizing antibody was detected in the serum and oral fluid samples post-inoculation suggesting antibody secretion (Pickering et al. 2021). The contradicting outcome of the experimental studies can be attributed to the difference in age, breed, viral isolate, and infectious dose (Meekins et al. 2020; Schlottau et al. 2020; Shi et al. 2020; Pickering et al. 2021). Pickering et al. (2021) used a higher viral dose (10-fold) for experimental inoculation than the previous studies. SARS-CoV-2 can infect and replicate in porcine kidney (PK-15) and swine testicle (ST) cell lines. Infection with SARS-CoV-2 was associated with cytopathic effects (CPE). However, experimental inoculation in five-week-old pigs did not result in infection (Meekins et al. 2020). Therefore, further studies are required to confirm the susceptibility of pigs to SARS-CoV-2 infection.

A serological survey was conducted to detect the presence of SARS-CoV-2 by using a commercial double-antigen sandwich ELISA. The test was used to detect the presence of SARS-CoV-2-specific antibodies among domestic livestock (cow, horse, sheep, and pig), poultry (chicken, duck, and goose), companion animals (dog and cat), and experimental animals (mice, rat, rabbit, guinea pig, and monkey) (Deng et al. 2020). However, none of the samples tested positive for SARS-CoV-2-specific antibodies. Another large-scale study was conducted among companion animals (603 dogs and 316 cats) living in northern Italy to assess the occurrence of SARS-CoV-2 infection. Among the sampled population, none of them were tested positive in real-time RT-PCR test. However, a small proportion of the dogs (3.3%) and cats (5.8%) had measurable SARS-CoV-2 specific neutralizing antibody titers. The dogs from COVID-19 positive households were found to have more chance to test positive for SARS-CoV-2 specific neutralizing antibodies than those from COVID-19 negative households (Patterson et al. 2020). Another large-scale serosurvey was conducted among dogs and cats in Croatia using ELISA and an in-house microneutralization test. The study confirmed the presence of neutralizing antibodies in 0.76% cats and 0.31% dogs (Stevanovic et al. 2020).

SARS-CoV-2 infected humans have sporadically been reported to transmit the virus to susceptible animals that are in close contact (reverse-zoonotic transmission) (Yoo and Yoo 2020). Cats and dogs are the two companion animal species that are often in close contact with human beings. Two dogs were tested positive for SARS-CoV-2 in Hong Kong SAR. Analysis of the genomic sequence suggested that the two dogs got the virus from their owners, who also tested positive for COVID-19 (Sit et al. 2020). Natural SARS-CoV-2 infection has been reported in domestic animal species such as dog, cat, ferret and mink (Mahdy et al. 2020; OIE 2020).

Cats are highly susceptible to subclinical infection, with a prolonged period of oral and nasal viral shedding that is generally not accompanied by clinical signs, and can transmit the infection to other cats through direct contact or aerosols (Bosco-Lauth et al. 2020, Gaudreault et al. 2020; Lakdawala and Menachery 2020, Shi et al. 2020). Two small scale serological investigations have been conducted in the domestic cat populations till now, one in China and another one in France (Temmam et al. 2020; Zhang et al. 2020). In Wuhan, China, where the virus emerged, 15% of cat serum samples collected after COVID-19 onset were positive for antibodies against the RBD of SARS-CoV-2 and no serological cross-reactivity was detected between the SARS-CoV-2 and type I or II feline infectious peritonitis coronavirus (FIPV) (Zhang et al. 2020). Another serological survey was conducted in France that screened a total of 9 cats that were living in close contact with their COVID-19 positive owners. Even though the owners were tested positive for COVID-19, none of the cats was infected as evidenced by negative RT-PCR and serological test results (Temmam et al. 2020). Since it is challenging to obtain a general conclusion from these serological surveys, further detailed investigations are required. The present situation also warrants the need for large scale serological surveys among the cat population to get an insight on the infectivity of SARS-CoV-2 under natural conditions.

**Figure 2.**
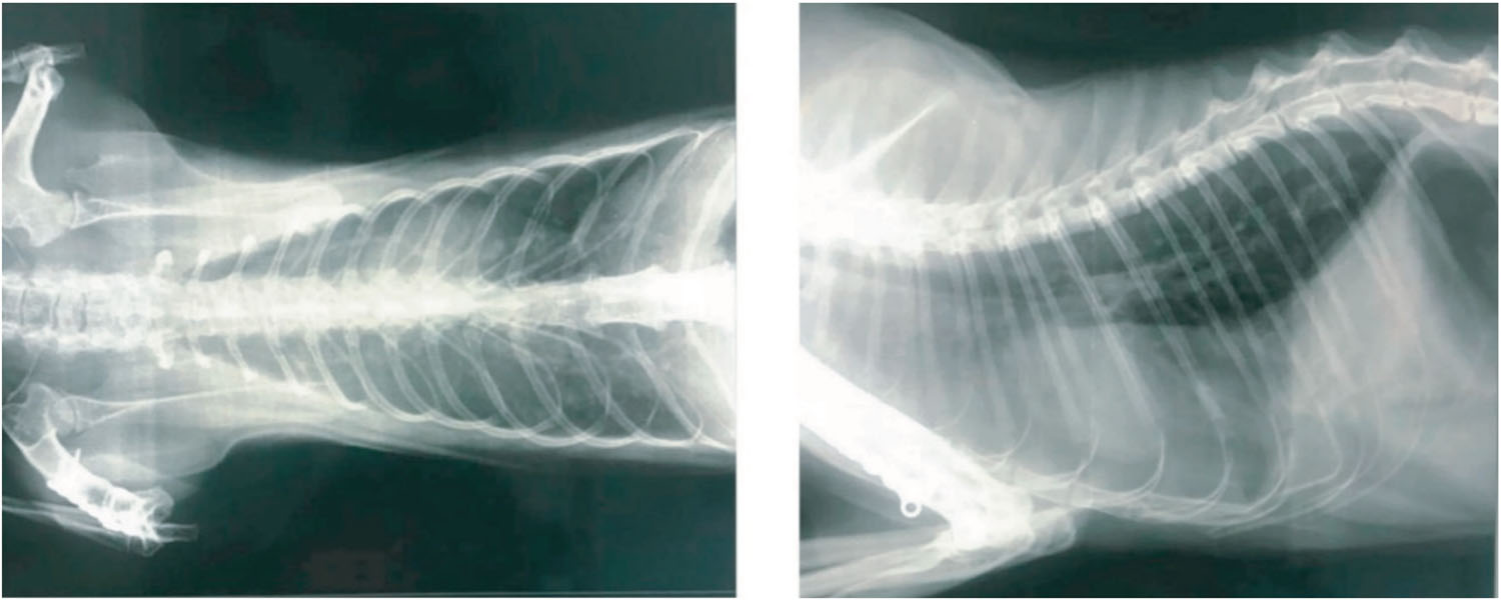
SARS-CoV-2 infection in an Italian pet cat. The thoracic radiographs revealed the presence of pneumonia. The opaque region in the radiograph over the lung area represents the ROI (Region of Interest). The widespread presence of pathological infiltrate resulted in an unstructured interstitial pattern. Reproduced from Musso et al. (2020) under Creative Commons Attribution (CC BY) license (http://creativecommons.org/licenses/by/4.0/.).

An Italian pet cat that exhibited clear clinical signs of pneumonia was diagnosed with SARS-CoV-2 infection as confirmed by quantitative RT–qPCR using deep pharyngeal swab. The lateral thoracic radiograph revealed the presence of an unstructured interstitial pattern associated with the presence of pathological infiltrates in the lung interstitium. Furthermore, the radiodensity of the lung parenchyma was found to be increased (Figure 2) giving a “ground glass” appearance (Musso et al. 2020). The genome sequence of the SARS-CoV-2 isolated from the infected cat in France belongs to the phylogenetic clade A2a. This is similar to those infecting French humans, suggesting transmission from human-to-cat (Sailleau et al. 2020). During the 2003 SARS outbreak, cats living near SARS positive human beings were also tested positive for SARS-CoV (Martina et al. 2003). However, there was no evidence of cat-to-human transmission for SARS-CoV. Considering the potential for trans-species transmission events between cats and human beings, a large-scale serological survey was conducted among the domestic cat population of Germany to assess the incidence of SARS-CoV-2. Among the 920 of serum samples evaluated using indirect multispecies ELISA and indirect immunofluorescence test, 0.69% (6/920) of the samples were found to be positive for SARS-CoV-2 specific antibodies (Michelitsch et al. 2020). Although there is strong evidence for human-to-cat transmission (Martina et al. 2003; Sailleau et al. 2020), the available evidence is not sufficient enough to confirm SARS-CoV-2 circulation in domestic cats as a result of cat-to-cat transmission even though they were found to be highly susceptible under experimental conditions.

Experimental studies have also identified that the transmissibility of SARS-CoV-2 is dramatically attenuated upon serial passaging in cats (via one-to-one homosexual cohousing of an infected cat with a naive cat). The cat-to-cat transmissibility of SARS-CoV-2 was found to be reduced over time in cats as compared to the sustained human-to-human transmission (Bao et al. 2021). This can be attributed to the variations existing in the binding sites of feline ACE2 receptor as compared to human beings (Bao et al. 2021). In addition to that, cats previously infected with SARS-CoV-2 were found to be susceptible to reinfection in the subsequent experimental inoculation. However, the degree of viral shedding was not sufficient enough to facilitate the transmission of SARS-CoV-2 to the co-housed naïve cats (Gaudreault et al. 2020). The successful establishment of cat-to-human transmission is a major problem as we have to exclusively rely on the evidence from real-world situations (Totton et al. 2021). Furthermore, human-to-human transmission is considered the main route of SARS-CoV-2 transmission making it difficult to provide definitive evidence of sporadic cat-to-human transmission. Therefore, it is possible that the direct evidence of cat-to-human transmission will never be obtained even if it occurs sporadically in the future.

Recent findings indicate that companion animals such as dogs and cats are also susceptible to SARS-CoV-2 variants identified in human beings (Ferasin et al. 2021; Gauntt 2021; Grimm 2021). The first known cases of SARS-CoV-2 infection with United Kingdom variant (B.1.1.7) was reported in a domestic shorthair cat and a black lab-mix dog from the same household in Texas, United States (Gauntt 2021). The owner was previously tested positive for COVID-19 following which the pets were tested. Although the pets did not exhibit any sign of illness at the time of their positive tests, both of them started to sneeze several weeks later (Gauntt 2021). Similarly, two cats and a dog were tested positive for SARS-CoV-2 B.1.1.7 variant in United Kingdom (Ferasin et al. 2021). The owners of these pets were diagnosed with COVID-19 and exhibited respiratory symptoms 3-6 weeks before their pets became ill. However, none of the pets exhibited respiratory signs, instead developed atypical clinical manifestations, including cardiac abnormalities secondary to myocarditis (Ferasin et al. 2021). Although the authors have hypothesized that the SARS-CoV-2 variant (B.1.1.7) is responsible for the occurrence of myocarditis in pets, there is not sufficient proof to substantiate such a claim. From the available data, it is evident that companion animals such as dogs and cats are susceptible to infection with SARS-CoV-2 variants. However, the impact of such variants in terms of transmissibility, disease severity, and pathogenesis are unknown (Grimm 2021). It is also unclear whether the B.1.1.7 variant is more transmissible between humans and animals compared to the original strain (Ferasin et al. 2021; Grimm 2021). Therefore, further studies are required to evaluate whether the newly emerged SARS-CoV-2 variants are more transmissible or dangerous in animals.

If future research proves that the virus can transmit from cats to humans, this may motivate people to abandon their cats for fear of infection (Rodriguez-Morales et al. 2020b; Gao et al. 2020). It has also been speculated that cats and other mammals could eventually become maintenance hosts for SARS-CoV-2 after spillover, thereby contributing to complicate the epidemiological picture (Bosco-Lauth et al. 2020, Franklin and Bevins 2020). The ease of SARS-CoV-2 transmission from infected cats to co-housed naïve cats is a matter of concern in animal shelters or dense urban stray cat colonies since as it will provide an ideal condition similar to the experimental one for disease transmission (Halfmann et al. 2020). Feral cats found in the surroundings of mink farms in the Netherlands were tested for the presence of SARS-CoV-2 infection. Among the 24 animals screened, seven were having antibodies against SARS-CoV-2, and one cat was tested positive for viral RNA (Oreshkova et al. 2020). These feral cats roam around the farms, stealing food and might have got the infection from an infected mink. Such stray/feral cats pose significant threats to their counterparts living in the wild. Feral cats do contact other, often endangered, wild carnivores, constituting a risk of infection transmission to valuable wildlife populations (López et al. 2009). In another seroprevalence study conducted among the stray cats captured in the city of Zaragoza (Spain), the presence of SARS-CoV-2 specific antibodies (IgG specific for RBD) was detected using an indirect ELISA (Villanueva-Saz et al. 2021). Among the 114 cats evaluated, four (3.51%) were seropositive SARS-CoV-2 specific antibodies. Furthermore, three among the four SARS-CoV-2 seropositive cats were also found to be seropositive to *Toxoplasma gondii* and feline immunodeficiency virus (FIV) (n = 1); *T. gondii* (n = 1); and FIV and *Leishmania infantum* (n = 1). The existence of concomitant infections with other pathogens (*T. gondii*, *L. infantum* and FIV) suggest that the immunosuppressed animals might have increased susceptibility to SARS-CoV-2 infection (Villanueva-Saz et al. 2021). Thus, the probability of stray cats transmitting SARS-CoV-2 to wild felids is high. Additionally, recent results have shown that coronaviruses are present in cat flea, *Ctenocephalides felis* (Villar et al. 2020). Hence, the role played by cats, including stray urban and feral cats, in the COVID-19 pandemic should be further investigated (Li 2020). However, there is no indication of long-term maintenance of SARS-CoV-2 infection in cat populations in the absence of re-infections from human beings.

Although current evidence suggests a low risk for domestic and companion animal-to-human transmission, the possibility of domestic animals acting as viral reservoirs for SARS-CoV-2 cannot be ruled out (Csiszar et al. 2020; Hernández et al. 2020; Hobbs and Reid 2020; Irian 2020; Mazinani and Rude 2021). This calls for the implementation of One Health strategies that involve expanding ongoing epidemiological surveillance to relevant animal populations (Bhatia 2020; Leroy et al. 2020; Bonilla-Aldana et al. 2020a).

## 7. SARS-CoV-2 infection in mustelids and implications to public health

**Figure 3.**
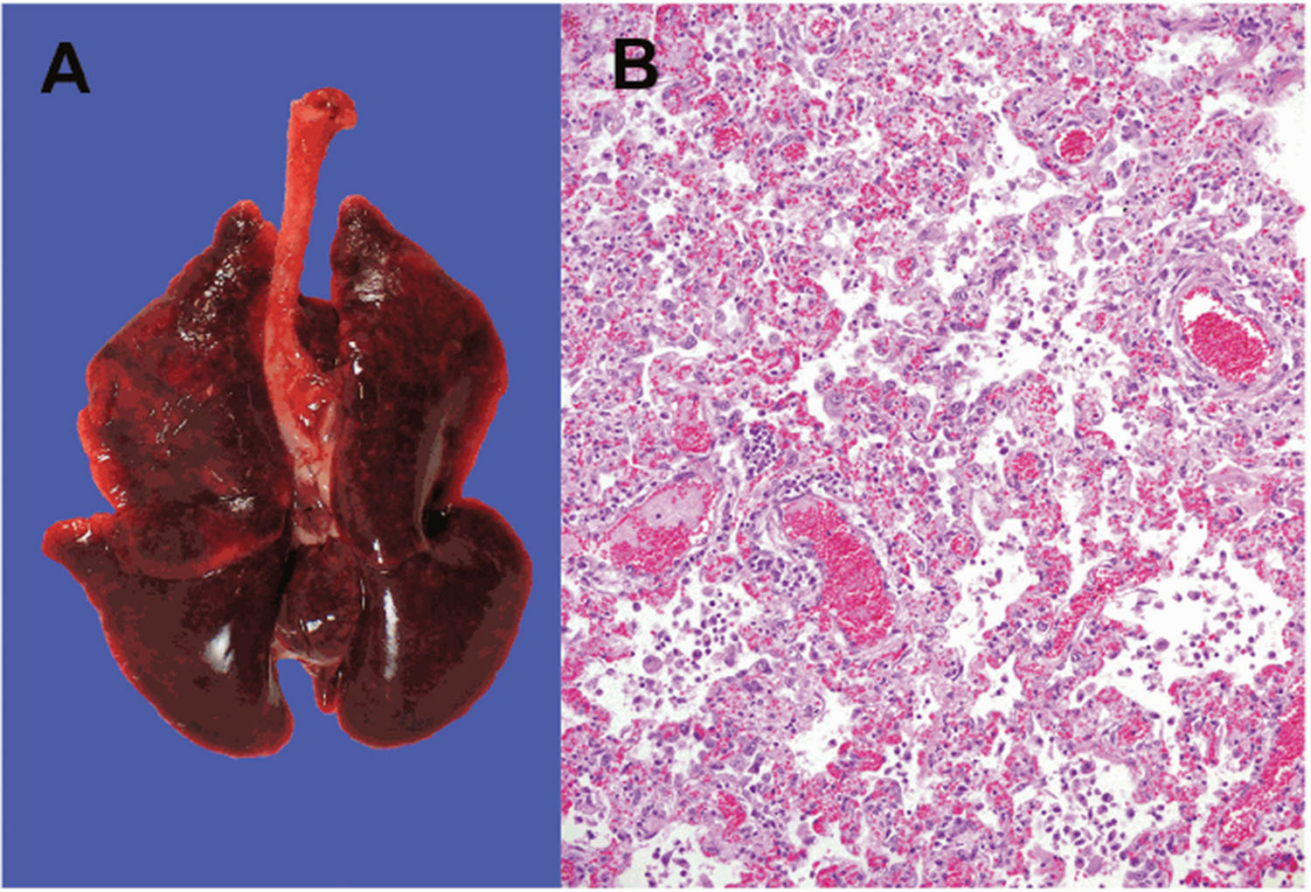
SARS-CoV-2 infection in farmed minks. **A** - Macroscopic image of the infected lungs. **B** - Microscopic image of 10% formalin fixed histopathological lung section stained with haematoxylin and eosin showing interstitial pneumonia (objective 20×). Reproduced from Oreshkova et al. (2020) under Creative Commons Attribution (CC BY 4.0) Licence (http://creativecommons.org/licenses/by/4.0/.).

Genetic and epidemiological investigations identified the occurrence of mink-to-human transmission in at least two workers of a mink farm who have caught the virus from minks. This was the first case of suspected animal-to-human transmission of SARS-CoV-2 reported in the world (Enserink 2020; Oude Munnink et al. 2020), but air-borne transmission could be discarded. In Denmark, the novel SARS-CoV-2 variants that were initially identified in minks subsequently appeared within the local human population, presumably by the initial mink-to-human transmission followed by human-to-human transmission (Hammer et al. 2020). The mink infected with SARS-CoV-2 exhibited signs of respiratory disease, and an increased mortality rate was reported on the mink farm (Oreshkova et al. 2020). SARS-CoV-2 can get transmitted among the mink population rapidly. It is suspected to be transmitted via infectious droplets, on feed or bedding, or faecal matter dust (Enserink 2020). The infected minks were having typical pathological findings of viral pneumonia that is observed in COVID-19 positive humans (Oreshkova et al. 2020). Histopathological analysis of the haematoxylin and eosin-stained lung sections was indicative of diffuse interstitial pneumonia and alveolar damage (Figure 3) (Oreshkova et al. 2020). Considering the possibility of mink developing into a viral reservoir of SARS-CoV-2, authorities in the Netherlands decided to put a ban on mink farming into action earlier than original due to the mink-human transmission. The susceptibility of mink to SARS-CoV-2 was not a surprise, as they are closely related to ferrets that were already found to be susceptible in experimental inoculation (Enserink 2020; Richard et al. 2020; Shi et al. 2020).

Following the SARS-CoV-2 outbreak in the mink farms of the Netherlands, U.S. Department of Agriculture (USDA) has confirmed SARS-CoV-2 outbreak on mink farms of United States of America that resulted in large-scale morality (Cahan 2020). In addition to the Netherlands and United States of America, minks are reported to be infected with SARS-CoV-2 in Denmark, Greece, France, Italy, Canada, Poland, Lithuania, Spain, and Sweden (Cahan 2020; OIE 2020; Oreshkova et al. 2020; WHO 2020). SARS-CoV-2 infection in mink is associated with respiratory disease. Histologic examination of the lung samples collected from the affected mink identified changes suggestive of acute interstitial pneumonia with extensive alveolar damage (Molenaar et al. 2020; Oreshkova et al. 2020). Although the minks were housed in separate cages separated by non-permeable partition, SARS-CoV-2 was found to spread rapidly (Cahan 2020). Considering all the factors, we can say that SARS-CoV-2 infection in mink is similar to COVID-19 in humans and mimics the clinical and pathological findings. Furthermore, the detection of SARS-CoV-2 RNA in the inhalable dust collected from the farm confirms the possible air-borne mink-to-mink, and mink-to-human transmission (Oreshkova et al. 2020; Zhao et al. 2020a).

SARS-CoV-2 infection in mink is posing a risk for humans since the rapid transmission among the mink population fueled the accumulation of mutations that further contributed to the evolution of virus (Oude Munnink et al. 2020). The World Health Organization (WHO) has reported the emergence of a mink-associated SARS-CoV-2 variant (WHO 2020). The new variant is associated with a series of mutations that are not yet reported elsewhere. The clinical presentation and disease severity of the new variant, referred to as the “cluster 5”, is similar to the circulating virus (WHO 2020). However, the impact produced by the new mink-associated variant on the sensitivity of diagnostic tests and the efficacy of vaccines and therapeutics that are currently under development are yet to be determined (Sharun et al. 2021). People in Denmark and the Netherlands are already infected with a SARS-CoV-2 mutant (Y453F) originated from minks. The mutation was found to be associated with a change in the spike protein amino acid (Mallapaty 2020b). A preliminary study using three-dimensional protein structural analysis suggested that the SARS-CoV-2 mutant (Y453F) can partially escape from the neutralizing monoclonal antibodies that are commercially available (CC12.1, CC12.3, CV07-250, and COVA2-04) (Hayashi et al. 2020). This can be attributed to the weak recognition of spike glycoprotein by the neutralizing monoclonal antibodies (Hayashi et al. 2020; Mallapaty 2020b). However, the analysis using an ELISA-based ACE2/RBD inhibition assay identified that the Y453F residue change does not decrease the established humoral immunity from previously infected individuals. Similarly, the Y453F variant does not affect the neutralizing antibody response in a vaccine mouse model based on the original Wuhan strain RBD as antigens (Bayarri-Olmos et al. 2021). Instead, the Y453F variant binds to the human ACE2 receptor with a four-fold higher affinity suggesting the potential for enhanced transmission thereby limiting our ability to control the viral spread (Bayarri-Olmos et al. 2021). Therefore, further studies are necessary to confirm the impact of mink-associated variants on the efficacy of vaccine and therapeutics. The mink-derived SARS-CoV-2 sequences isolated from the Netherlands and Denmark contained multiple substitutions in the RBD of Spike protein. Further analysis confirmed that these substitutions increased the binding energy indicating adaptation of the Spike protein to the mink ACE2 receptor. These substitutions might also have an impact on the binding affinity of human ACE2 and the humoral immune responses directed against the Spike protein RBD (Welkers et al. 2021). The detection of ORF8-deficient lineages with N501T spike mutation in farmed mink and humans from Denmark suggest that lineages without ORF8 can undergo spillover from one species to another. The loss of ORF8 might have occurred as a result of rapid SARS-CoV-2 transmission within the mink population (Pereira 2021). The identification of mink-associated SARS-CoV-2 variant highlights the danger of having viral reservoirs for SARS-CoV-2.

Mink is the only free-living wildlife that has been infected with SARS-CoV-2. The asymptomatic infection was confirmed by real time RT-PCR and sequencing using the nasal swab sampled in Utah (ProMed 2020). The infected mink was identified during the survey conducted among the free-roaming mammals (78 rodents and 24 mesocarnivores) captured on and around the mink farms previously affected with SARS-CoV-2 outbreak. The sampled rodents included rock squirrels (*Otospermophilus variegatus*), *Peromyscus* spp. mice, deer mice (*Peromyscus maniculatus*), and house mice (*Mus musculus*) whereas the mesocarnivores included escaped and wild American minks (*Neovison vison*), striped skunks (*Mephitis mephitis*), and raccoons (*Procyon lotor*) (Shriner et al. 2021). Although at present there is no solid evidence to substantiate that SARS-CoV-2 is circulating or has been established in wild animal populations living in close proximity to the infected mink farms, the possibility of such a scenario cannot be ruled out. Furthermore, the viral genome obtained from the wild mink was indistinguishable from the ones obtained from the farmed mink (ProMed 2020). The escapees from the mink farms have the potential to establish self-sustaining population in the wild. Therefore, strict measures have to be implemented to prevent the introduction and subsequent spread of SARS-CoV-2 within the wild mink population.

**Figure 4.**
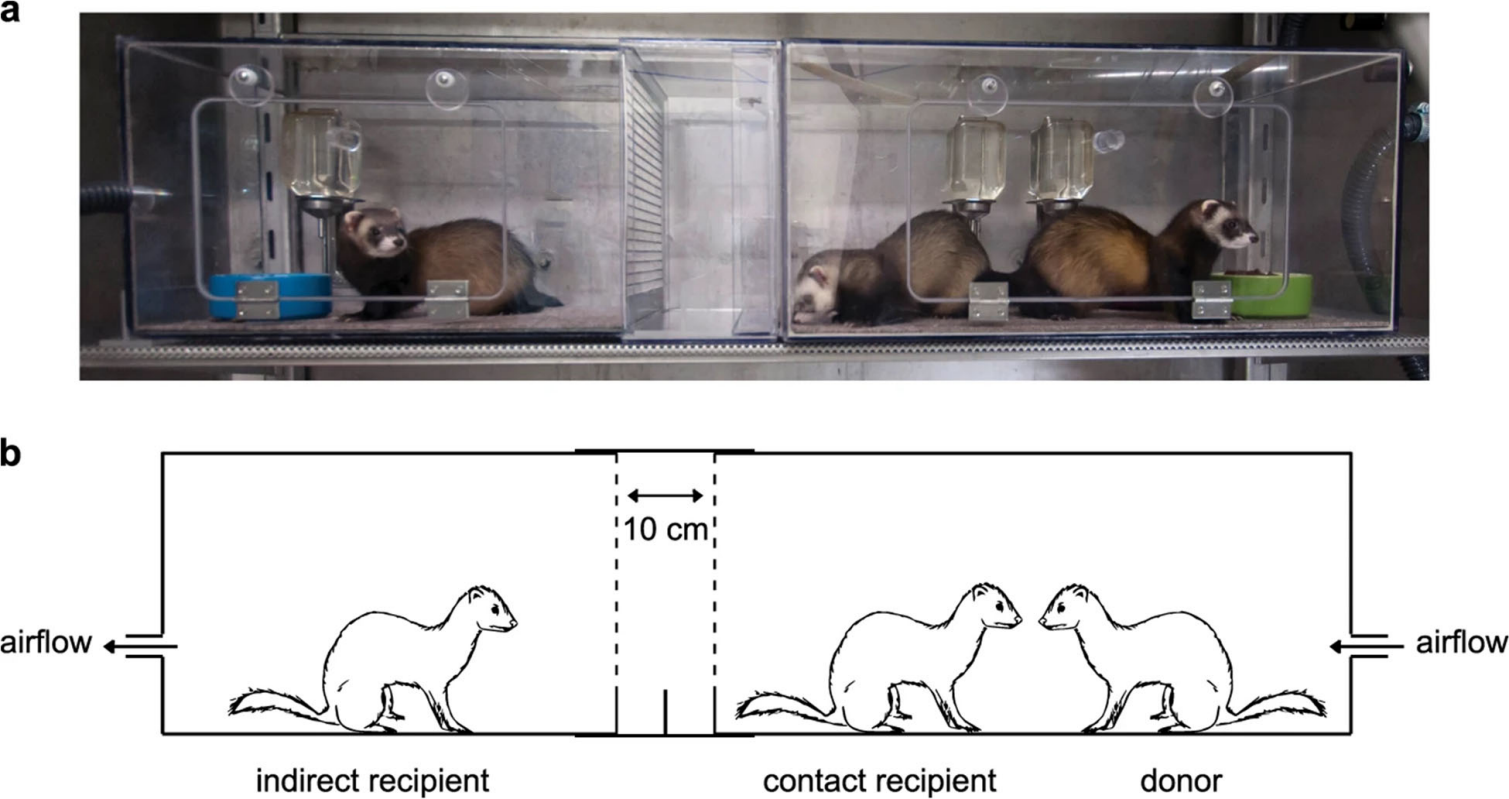
**(a and b)** The experimental set up to establish direct transmission (via contact) and indirect transmission (via air) of SARS-CoV-2 in ferrets. The inoculated donor ferret is kept in a cage while the recipient ferret is placed in another cage that is separated by two steel grids, 10 cm apart, to prevent direct contact. Reproduced from Richard et al. (2020) under Creative Commons Attribution 4.0 International License (http://creativecommons.org/licenses/by/4.0/.).

Experimental inoculation of SARS-CoV-2 in ferrets resulted in a mild clinical disease that can be transmitted from infected to susceptible animals via direct contact (Richard et al. 2020; Shi et al. 2020) and via air (Richard et al. 2020). The evidence of direct (via contact) and indirect (via air) SARS-CoV-2 transmission was obtained by performing an experiment that involves housing an inoculated donor ferret with a direct contact ferret and an indirect recipient ferret (Figure 4). Infected ferrets shed virus in saliva, nasal washes, feaces, and urine up to 8 days post-infection (Kim et al. 2020). In a comparative experimental study involving the inoculation of ferrets using high or medium dose of SARS-CoV-2, high dose intranasal challenge was found to be associated with better viral RNA shedding via the upper respiratory tract (Ryan et al. 2021). Furthermore, the recovered ferrets (after the cessation of viral shedding) were found to be fully protected from acute lung pathology in the subsequent inoculation (Ryan et al. 2021).

Importantly, large numbers of ferrets are kept in some countries, notably Spain, for rabbit hunting and rabbit control purposes. Some of these ferrets do eventually escape and establish feral populations (Medina and Martín 2010). A sporadic case of SARS-CoV-2 infection was reported in a kept pet ferret from an infected household in Slovenia (OIE 2020). Another investigation was conducted in ferrets that are used as working animals for rabbit hunting. SARS-CoV-2 RNA was detected in the oropharyngeal and rectal swabs collected from 6 of 71 ferrets (8.4%) using a RT-qPCR assay. SARS-CoV-2 was also isolated from the rectal swab collected from one of the ferrets (Gortazar et al. 2021). In another study, the presence of SARS-CoV-2 specific antibodies were evaluated in the household ferrets sampled in Spain using an in-house ELISA based on spike protein RBD (Giner et al. 2021). Among the 127 ferrets screened, only two (1.57%) tested positive for the presence of SARS-CoV-2 antibodies. The anti-RBD antibodies persisted for more than 129 days since its first detection in the domestic ferret (Giner et al. 2021). Ferrets are considered pets in some parts of the world. Furthermore, they are also used as hunting/working animals for rabbit control (Gortazar et al. 2021). Considering the susceptibility of rabbits to SARS-CoV-2 under experimental condition (Mykytyn et al. 2021), further studies are required to investigate the potential of interspecies transmission of SARS-CoV-2 from infected ferrets to wild rabbits during hunting. Both mink and ferrets belong to the family Mustelidae. Therefore, the pattern of infection suggests that other members of the family Mustelidae may be susceptible to SARS-CoV-2 and therefore require further investigation.

## 8. SARS-CoV-2 in zoo and wild animals

Several reports of SARS-CoV-2 infection in wild animal species have been documented (Delahay et al. 2020; Franklin and Bevins 2020; Leroy et al. 2020; Martínez-Hernández et al. 2020; Tiwari et al. 2020). On 4^th^ April 2020, the first case of COVID-19 in zoo animals was confirmed in a Malayan tiger maintained at the Bronx Zoo, New York (Centers for Disease Control and Prevention 2020). The samples were taken for testing when several big cats started showing signs of respiratory illness. This was followed by another four tigers and three lions from the same zoo, which tested positive for COVID-19, increasing the total number of SARS-CoV-2 positive zoo animals to eight (National Geographic 2020). These captive wild felids were suspected to be infected from a COVID-19 positive zookeeper/worker. The whole-genome sequence of SARS-CoV-2 isolated from the Malayan tiger (SARS-CoV-2/tiger/NY/040420/2020) was consistent with the available SARS-CoV-2 sequences from humans (Wang et al. 2020b). Later, a case in a puma was reported from a zoo in South Africa after having contact with an infected handler (OIE 2020). A female snow leopard that exhibited very mild symptoms was later tested positive for SARS-CoV-2 at the Louisville Zoo (Louisville Zoo 2020).

A serological survey was also conducted among wild animals for detecting the presence of SARS-CoV-2-specific antibodies. However, none of the samples tested positive for SARS-CoV-2-specific antibodies (Deng et al. 2020). As the number of COVID-19 cases is on the rise, spillover of this pandemic virus to susceptible wild animal populations is likely to occur. Recent reports of SARS-CoV-2 in Malayan tiger, lion, and domestic cat suggest that the virus can get transmitted to other felids (Wang et al. 2020b). Considering the susceptibility of the Felidae family, the transmission of SARS-CoV-2 to the wild animal population might have already taken place. Among the 103 animal species that exhibited very high, high and medium interaction between ACE2 and SARS-CoV-2 RBD, 41 species (40%) come under the ‘Threatened’ categories of IUCN Red List (Vulnerable, Endangered, and Critically Endangered) (Damas et al. 2020). Another group of species of particular concern is great apes since these are endangered and share many characteristics with humans (Gillespie and Leendertz 2020). The western lowland gorillas at the San Diego Zoo Safari Park also tested positive for SARS-CoV-2. All the exposed gorillas in the troop have exhibited mild respiratory symptoms such as coughing and congestion. The gorillas are believed to have acquired the infection from an asymptomatic member of the San Diego Zoo Safari Park’s wildlife team (Gibbons 2021). One of the infected gorillas developed pneumonia and heart disease due to advanced age and underlying medical conditions. The gorilla was treated using antibiotics, heart medications, and a cocktail of monoclonal antibodies thereby resulting in complete recovery (San Diego Zoo Global 2021). Therefore, the implementation of mandatory vaccination along with the establishment of a treatment protocol constituting of antiviral drugs and SARS-CoV-2 specific monoclonal antibodies will help to control and prevent SARS-CoV-2 infection in great apes. Some of the national parks in Africa were locked down as an effort to protect the population of great apes (Mongabay 2020). The Virunga National Park in the Democratic Republic of Congo hosts one of the last remaining populations of the mountain gorilla (*Gorilla beringei beringei*). SARS-CoV-2 infection in such threatened and endangered animal populations could have serious consequences and therefore has to be avoided at all cost.

Recently, white-tailed deer was found to be highly susceptible to SARS-CoV-2 infection following experimental infection. Intranasal inoculation in the deer fawns resulted in the establishment of subclinical viral infection along with viral shedding via nasal secretions and feaces. The infected deer was also able to transmit the virus to indirect contact animals (Palmer et al. 2021). Considering the high susceptibility of white-tailed deer to SARS-CoV-2 infection, further investigations are warranted among the wild cervid species to identify the potential reservoir hosts of SARS-CoV-2. The susceptibility of fruit bats (*Rousettus aegyptiacus*) to SARS-CoV-2 infection was evaluated under experimental conditions. Among the inoculated fruit bats, 78% had a transient infection that was detected using RT-qPCR and immunohistochemistry. Furthermore, SARS-CoV-2 RNA was also detected in the lung, lung-associated lymphatic tissue, and trachea (Schlottau et al. 2020). The infected fruit bats were also able to transmit the virus to contact bats resulting in infection (Schlottau et al. 2020). Although the findings from this study point towards the potential of fruit bats to serve as a reservoir host model, the risk of SARS-CoV-2 infection in free-living bats will have a great impact on the ecological bat protection programs.

The increasing reports of SARS-CoV-2 infection in animals including wildlife species may pose a potential risk to a broad range of mammals, and this should be considered as a serious issue since it has significant implications for threatened wild animal species as well as those under human care (including work and zoo animals). More investigations are needed with regards to risk assessment analysis of SARS-CoV-2 in wildlife population, enhancing wildlife surveillance and monitoring, exploring animal spillover events, reverse-zoonotic transmission risk and zoonotic implications for this pandemic virus, as well strengthen the implementation of integrated one health approach for safeguarding health of humans, animals and wild animals. Appropriate measures are warranted for avoiding and/or limiting human contacts with wild animals, adopting preventative strategies to protect wildlife researchers, professionals and workers, follow proper disposal as well as regular monitoring of waste materials and sewage that could potentially contaminate the environment, and design suitable mitigation strategies to counter COVID-19 pandemic (Barbosa et al. 2021 Delahay et al. 2020; Dhama et al. 2021; Martínez-Hernández et al. 2020).

**Figure 5.**
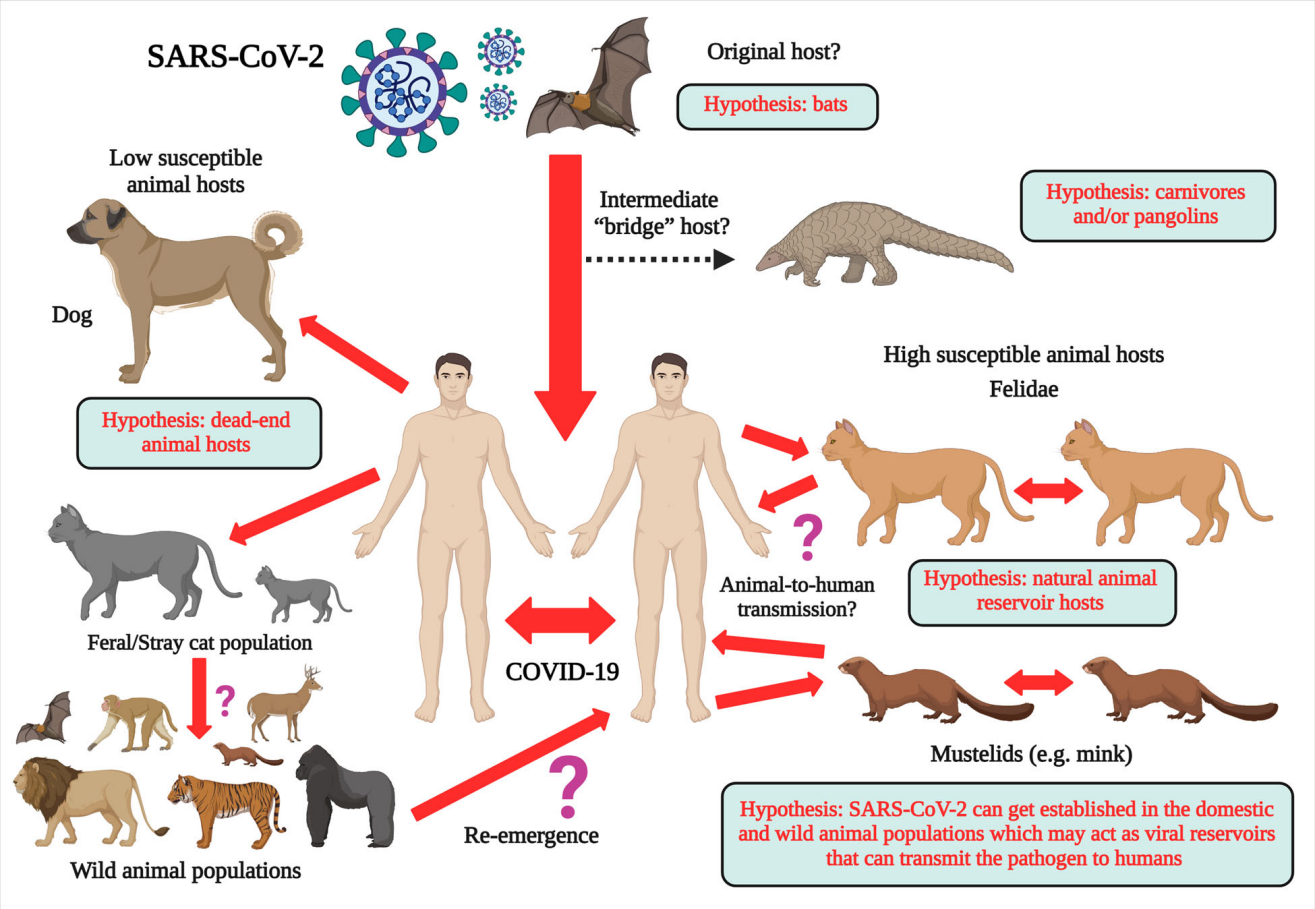
Illustration of some of the established and hypothesized transmission routes of SARS-CoV-2 between humans, domestic and wild animals.

Some of the established and hypothesized transmission routes of SARS-CoV-2 between humans, domestic and wild animals are illustrated in Figure 5. The susceptibility of different animal species to SARS-CoV-2 infection based on natural infection, experimental infection and genome analysis are described in Table 1.

**Table 1.** Susceptibility of different animal species to SARS-CoV-2 infection based on natural infection, experimental infection and genome analysis.

Animal species	Study type	Susceptibility	Transmission	Clinical signs	References
Dog (*Canis lupus familiaris*) (Natural infection reported in several countries)	Natural and experimental infection	Low	No	No	Shi et al. 2020; Sit et al. 2020
Cat (*Felis catus*) (Natural infection reported in several countries)	Natural and experimental infection	High	Yes, between cats	Yes (none to mild signs)	Shi et al. 2020; Sailleau et al. 2020
Malayan tiger (*Panthera tigris* subsp. *jacksoni*) (Natural infection reported in Bronx Zoo, New York, USA)	Natural infection	High	Not confirmed	Yes	Wang et al. 2020b; OIE. 2020
Lion (*Panthera leo*) (Natural infection reported in Bronx Zoo, New York, USA)	Natural infection	High	Not confirmed	Yes	OIE. 2020
Puma (*Puma concolor*) (Natural infection reported in Gauteng Zoo, Johannesburg, South Africa)	Natural infection	High	Not confirmed	Yes	OIE. 2020
Snow leopard (*Panthera uncia*) (Natural infection reported in Louisville Zoo, Kentucky, USA)	Natural infection	High	Not confirmed	Yes	OIE. 2020
American mink (*Neovison vison*) (Natural infection reported in Denmark, Greece, Italy, France, Canada, Lithuania, Spain, Poland, Sweden, The Netherlands and USA)	Natural infection	High	Yes, between minks and mink- to-human	Yes	Cahan 2020; Oreshkova et al. 2020; Oude Munnink et al., 2020
Ferret (*Mustela putorius furo*)	Experimental and natural infection	High	Yes, between ferrets and via air	No (very mild in some)	Shi et al. 2020; Kim et al. 2020; Richard et al. 2020; OIE. 2020
Rabbits (*Oryctolagus cuniculus*)	Experimental infection	Yes	No	No	Mykytyn et al., 2020
Pig (*Sus scrofa domesticus*)	Experimental infection	No	No	No	Shi et al. 2020
Raccoon dogs (*Nyctereutes procyonoides*)	Experimental infection	Yes	Yes, between raccoon dogs	Subtle clinical signs	Freuling et al. 2020
North American raccoons (*Procyon lotor*)	Experimental infection	Yes	No	No	Francisco et al., 2020
Striped skunks (*Mephitis mephitis*)	Experimental infection	Yes	No	No	Francisco et al., 2020
Chicken (*Gallus gallus domesticus*)	Experimental infection	No	No	No	Shi et al. 2020; Suarez et al. 2020
Duck (Anatidae)	Experimental infection	No	No	No	Shi et al. 2020
Pekin duck (*Anas platyrhinchos domesticus*)	Experimental infection	No	No	No	Suarez et al., 2020
Turkeys (*Meleagris gallopavo*)	Experimental infection	No	No	No	Suarez et al., 2020
White Chinese geese (*Anser cygnoides*)	Experimental infection	No	No	No	Suarez et al., 2020
Japanese quail (*Coturnix japonica*)	Experimental infection	No	No	No	Suarez et al., 2020
Egyptian fruit bats (*Rousettus aegyptiacus*)	Experimental infection	High	No	No	Friedrich-Loeffler-Institut 2020
Tree shrew (*Tupaia belangeris*)	Experimental infection	Low	No	No	Zhao et al. 2020b
Golden Syrian hamsters (*Mesocricetus auratus*)	Experimental infection	High	Yes, between hamsters	Yes (none to mild signs)	Sia et al. 2020
Chinese hamsters (*Cricetulus griseus*)	Experimental infection	High	Not confirmed	Yes	Bertzbach et al. 2020
Deer mice (*Peromyscus maniculatus*)	Experimental infection	High	Yes	Yes	Fagre et al. 2020
Rhesus macaque (*Macaca mulatta*)	Experimental infection	High	Yes	Yes	Lu et al. 2020; Munster et al. 2020
Common marmosets (*Callithrix jacchus*)	Experimental infection	Low	No	No	Lu et al. 2020
African green monkey (*Chlorocebus aethiops*)	Experimental infection	Yes	No	No	Woolsey et al. 2020
Cynomolgus macaque or Crab-eating macaque (*Macaca fascicularis*)	Experimental infection	High	Yes	Yes	Lu et al. 2020; Rockx et al., 2020
Western lowland gorilla (*Gorilla gorilla*) (Natural infection reported in San Diego Zoo Safari Park, USA)	Natural infection	High	Not confirmed	Yes	Gibbons 2021
White-tailed deer (*Odocoileus virginianus*)	Experimental infection	High	Yes	No	Palmer et al. 2021
Cattle (Bos taurus)	Experimental infection	Low	No	No	Ulrich et al. 2020
*Rhinopithecus roxellana* (golden snub-nosed monkey), *Macaca mulatta* (rhesus macaque), *Pan troglodytes* (chimpanzee), *Papio anubis* (olive baboon), *Callithrix jacchus* (common marmoset), *Pongo abelii* (Sumatran orangutan)	Genome analysis of ACE2 receptor	ACE2 can bind to S protein	Unknown	Unknown	Luan et al. 2020b
*Rhinolophus macrotis* (big-eared horseshoe bat), *Rhinolophus sinicus* (Chinese rufous horseshoe bat), *Rousettus leschenaultii* (Leschenault’s rousette), *Rhinolophus pearsonii* (Pearson’s horseshoe bat), *Pteropus vampyrus* (large flying fox)	Genome analysis of ACE2 receptor	ACE2 can bind to S protein	Unknown	Unknown	Luan et al. 2020b
*Sus scrofa* (wild boar), *Sus scrofa domesticus* (domestic pig), *Canis lupus familiaris* (dog), *Felis catus* (cat), *Equus caballus* (horse), *Bos taurus* (cattle), *Ovis aries* (sheep), *Vulpes* (red fox)	Genome analysis of ACE2 receptor	ACE2 can bind to S protein	Unknown	Unknown	Luan et al. 2020b
*Oryctolagus cuniculus* (rabbit), *Phodopus campbelli* (Campbell’s hamster), *Mesocricetus auratus* (golden hamster), *Heterocephalus glaber* (naked mole-rat), *Ictidomys tridecemlineatus* (thirteen-lined ground squirrel), and *Cricetulus griseus* (Chinese hamster), *Mustela erminea* (stoat), *Paguma larvata* (masked palm civet), *Mustela putorius furo* (ferret), *Manis javanica* (pangolin)	Genome analysis of ACE2 receptor	ACE2 can bind to S protein	Unknown	Unknown	Luan et al. 2020b
*Camelus dromedarius* (dromedary camel), *Loxodonta africana* (African elephant), *Procyon lotor* (raccoon), *Erinaceus europaeus* (European hedgehog), *Rhinolophus ferrumequinum* (greater horseshoe bat), *Nyctereutes procyonoides* (raccoon dog), *Suricata suricatta* (meerkat)	Genome analysis of ACE2 receptor	ACE2 cannot bind to S protein	Unknown	Unknown	Luan et al. 2020b
*Rattus norvegicus* (rat), *Mus musculus* (mouse), *Ornithorhynchus anatinus* (platypus), *Dipodomys ordii* (Ord's kangaroo rat), and *Cavia porcellus* (guinea pig)	Genome analysis of ACE2 receptor	ACE2 cannot bind to S protein	Unknown	Unknown	Luan et al. 2020b

### 8.1. SARS-CoV-2 vaccine for animals

Although companion animals such as dogs and cats are not likely to transmit SARS-CoV-2 to humans, experimental findings indicate the potential for cat-to-cat transmission (Shi et al. 2020; Sharun et al. 2020b). Even though animal-to-animal and animal-to-human transmission has only been reported in case of farmed minks under natural conditions, vaccination of domestic cats and dogs will help to prevent the spillover from humans. The LinearDNA™ COVID-19 vaccine candidate (Applied DNA Sciences and EvviVax) is an animal specific vaccine developed for use in cats. The vaccine has already received regulatory approval from United States Department of Agriculture (USDA) to undergo safety and immunogenicity evaluation in domestic felines (Brook 2020). The successful immunization using animal specific SARS-CoV-2 vaccine will help to prevent SARS-CoV-2 infection in felines.

Following the reports of SARS-CoV-2 infection in gorillas, the captive bonobos and orangutans at the San Diego Zoo in the USA became the first non-human primates to receive an experimental COVID-19 vaccine developed by Zoetis (Daly 2021). This experimental vaccine was specifically developed for use in dogs and cats. Its efficacy in dogs and cats has already been demonstrated in preliminary trials (Zoetis 2021). Zoetis is now in discussion with USDA for obtaining conditional approval for its use (Zoetis 2021). In addition to that, the COVID-19 vaccine candidates developed for humans can be utilized for inducing immunity in animals as they have already undergone in vivo evaluation in animal models such as ferrets, hamsters, and rhesus macaques (Sharun et al. 2020a). However, the antigen dose will need to be altered depending on the animal species undergoing vaccination.

SARS-CoV-2 has the potential to get transmitted from human-to-wildlife owing to the high susceptibility exhibited by primates, felids and mustelids (Sharun et al. 2020a). It is important to protect the captive wild animal species (especially those classified as endangered/threatened) that are kept under close proximity to humans (Gryseels et al. 2020). Therefore, the vaccination of captive wild animals (especially primates and felids) using animal specific SARS-CoV-2 vaccines will induce immunity and subsequently prevent spillover events in susceptible animal species.

## 9. Conclusion

For an animal species to act as a successful reservoir host, the virus has to get transmitted to the susceptible animal population from humans. Furthermore, it has to get established in the animal population with the help of efficient intra-species transmission. The animal species will only be called a reservoir host when it efficiently reintroduces the virus to human beings. Based on the current evidence, we can confirm that minks possess the potential to get established as a viral reservoir host of SARS-CoV-2. Recent evidence obtained from Denmark indicate that the SARS-CoV-2 introduced into the mink farms as a consequence of human-to-mink transmission has undergone viral mutations resulting in the emergence of mink-associated variant strains. This new SARS-CoV-2 strain got subsequently reintroduced to humans along with other emerging variants are generating a worldwide concern (Lauring and Hodcroft 2021). In addition to the potential for future spillover events, the emergence and circulation of new mink-associated variant strains in humans might enhance the transmissibility or virulence of SARS-CoV-2. Although several cases of mink-associated variant strains have been reported till now, the impact produced by such strains on vaccine and therapeutic efficacy are yet to be determined.

Considering the importance of animal coronaviruses and lessons learned from the ongoing COVID-19 pandemic with implications of animal spillover events, cross-species jumping and zoonotic concerns of SARS-CoV-2 (Jo et al. 2020; Kumar et al. 2020), it is time to take the One Health concept seriously, meaning that pathogen ecology and disease management need to integrate human, animal and environmental perspectives and follow necessary mitigation strategies to efficiently address health issues (Dhama et al. 2013; Bhatia 2020; Gortázar and de la Fuente 2020; Poudel et al. 2020; Bonilla-Aldana et al. 2020b). Based on the available evidence, it is logical to speculate about the possible occurrence of animal reservoirs of SARS-CoV-2 and other relevant Betacoronaviruses, as well as of eventual animal-to-human transmission as already suggested in mink (Delahay et al. 2020; Oreshkova et al. 2020). However, the “bridge” animal species have not been identified, and the lack of sound evidence for animal-to-human transmission with the only exception of mink suggests the possibility that viruses transmitted from humans to most animals lose the ability to re-infect human hosts. It is clear that for SARS-CoV-2, human-to-human transmission remains the main route. However, the fact that the virus infects and multiplies in animal hosts supports the need for animal surveillance to track virus prevalence and circulation and the possibility of spill-back to humans under still unknown conditions.

Furthermore, some of the animal species in which SARS-CoV-2 has been identified (cats, mustelids) have been infected with other CoVs, thus rolling out virus interference and antibody cross-protection. Considering the seriousness of the current pandemic situation and the potential impact caused by an animal reservoir in the transmission dynamics of SARS-CoV-2 in the human population, efficient surveillance systems have to be established for monitoring SARS-CoV-2 infection in wild and domestic animals. The increasing reports of SARS-CoV-2 in animals, especially in felids and mustelids, should be considered as a matter of concern in terms of animal health, welfare, and wildlife conservation. The recent developments in knowledge on animal susceptibility to SARS-CoV-2 call for the implementation of strict surveillance and routine screening in wild animals around the world.
